# MKP-1 Deficiency Exacerbates Skin Fibrosis in a Mouse Model of Scleroderma

**DOI:** 10.3390/ijms24054668

**Published:** 2023-02-28

**Authors:** Morena Scotece, Mari Hämäläinen, Tiina Leppänen, Katriina Vuolteenaho, Eeva Moilanen

**Affiliations:** The Immunopharmacology Research Group, Faculty of Medicine and Health Technology, Tampere University and Tampere University Hospital, 33014 Tampere, Finland

**Keywords:** scleroderma, fibrosis, inflammation, cytokines, cell signaling, MKP-1, DUSP1

## Abstract

Scleroderma is a chronic fibrotic disease, where proinflammatory and profibrotic events precede collagen accumulation. MKP-1 [mitogen-activated protein kinase (MAPK) phosphatase-1] downregulates inflammatory MAPK pathways suppressing inflammation. MKP-1 also supports Th1 polarization, which could shift Th1/Th2 balance away from profibrotic Th2 profile prevalent in scleroderma. In the present study, we investigated the potential protective role of MKP-1 in scleroderma. We utilized bleomycin-induced dermal fibrosis model as a well-characterized experimental model of scleroderma. Dermal fibrosis and collagen deposition as well as the expression of inflammatory and profibrotic mediators were analyzed in the skin samples. Bleomycin-induced dermal thickness and lipodystrophy were increased in MKP-1-deficient mice. MKP-1 deficiency enhanced collagen accumulation and increased expression of collagens, 1A1 and 3A1, in the dermis. Bleomycin-treated skin from MKP-1-deficient mice also showed enhanced expression of inflammatory and profibrotic factors IL-6, TGF-β1, fibronectin-1 and YKL-40, and chemokines MCP-1, MIP-1α and MIP-2, as compared to wild-type mice. The results show, for the first time, that MKP-1 protects from bleomycin-induced dermal fibrosis, suggesting that MKP-1 favorably modifies inflammation and fibrotic processes that drive the pathogenesis of scleroderma. Compounds enhancing the expression or activity of MKP-1 could thus prevent fibrotic processes in scleroderma and possess potential as a novel immunomodulative drug.

## 1. Introduction

Systemic sclerosis (SSc) is a chronic fibrosing autoimmune disease affecting skin and internal organs. Scleroderma is a skin manifestation of this disease, and it is typified by fibrotic changes resulting in thickening and hardening of skin. Although its etiology remains unknown, the early SSc is characterized by a combination of vasculopathy, autoimmunity and inflammation [1,2]. Activated fibroblasts produce excessive amounts of extracellular matrix (ECM) components, collagen and glycoproteins like fibronectin, which are accumulated in different organs causing variable disease subtypes [3,4]. Currently, there is no disease-modifying drug treatment for SSc. Treatment of organ-specific complications (renal crisis, pulmonary arterial hypertension and some other SSc organ manifestations) has improved survival, but SSc still has the highest cause-specific mortality of any of the rheumatic diseases, and especially patients with diffuse cutaneous systemic disease have a poor prognosis [1,2,5].

Imbalanced inflammation and activated dendritic cells play an important role in triggering fibrosis in SSc [1,2]. Type I interferon (IFN) signature, T helper (Th) 2 and alternative macrophage (M2-type) activation are characteristic features of inflammation preceding collagen accumulation [2]. Several lines of evidence suggest inflammatory cells as important sources of profibrotic mediators interleukin-6 (IL-6), transforming growth factor (TGF)-β1, fibronectin, YKL-40 and chemokines that initiate fibrotic processes through the activation of fibroblasts [6,7,8,9]. One of the feasible approaches is to target these key mediators or inflammatory signaling pathways that are involved in the pathogenesis of the disease [1,2,9].

Mitogen-activated protein kinase phosphatase-1 (MKP-1, also known as dual-specificity phosphatase 1, DUSP1), is a nuclear-localized phosphatase present in most cell types and tissues. MKP-1 is a negative feedback regulator of mitogen-activated protein kinase (MAPK) signaling pathways Erk1/2, p38, and c-Jun NH2-terminal kinase (JNK) that regulate many cellular responses such as growth, differentiation, mitosis and inflammatory response [10,11]. In vitro and in vivo studies have shown that MKP-1 is an important regulator of innate and adaptive immune responses and inflammation [12,13,14,15]. MKP-1 deficiency leads to a more severe disease in experimental arthritis and psoriasis [16,17]. Importantly, MKP-1 supports Th1 polarization by inducing interleukin-12 (IL-12) expression through interferon regulatory factor 1 (IRF1), which could be important in preventing Th2-supported fibrotic processes and development of scleroderma [18,19].

All these findings open an interesting possibility that MKP-1 could have a protective role in fibrosing diseases such as scleroderma and that hypothesis was approached in the present study. There is no perfect mouse model able to summarize every facet of scleroderma, but bleomycin-induced dermal fibrosis is a well-characterized experimental model used to evaluate the potential role of genes or treatments in the early inflammatory phase and/or in the subsequent fibrosis process typical for scleroderma [20]. We decided to investigate the potential protective role of MKP-1 in the pathogenesis of scleroderma by using the bleomycin-induced dermal fibrosis model in wild-type and MKP-1-deficient mice.

## 2. Results

### 2.1. Bleomycin-Induced Dermal Fibrosis Is Enhanced in MKP-1-Deficient Mice

**Figure 1:**
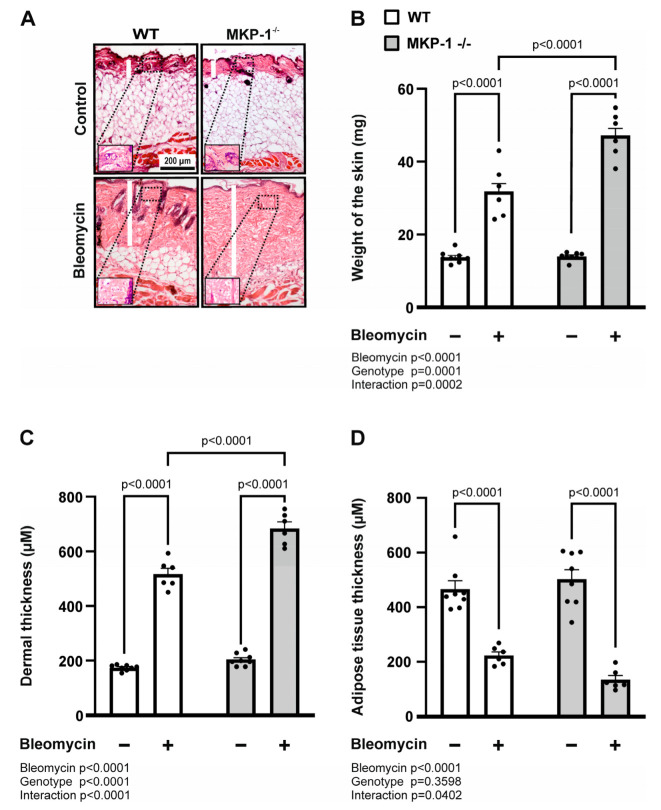
Dermal fibrosis is enhanced in bleomycin-treated MKP-1-deficient mice. (**A**) Representative images of H&E-stained sections of untreated control skin and skin treated with bleomycin from wild-type (WT) and MKP-1-deficient (MKP-1^−/−^) mice are shown. Original magnification 10×, magnification in insets 20×, scale bar 200 µm. (**B**) Skin samples (two 6 mm skin specimens per mouse) were harvested and weighted before proceeding to the histological analyses. (**C**) Dermal thickness (rectangular blank bar as shown in A, μm) was measured at six randomly selected locations in each section using the Image J program. (**D**) Thickness of the subcutaneous adipose tissue layer was measured by Image J program (six sites per mouse). Replicate measures were averaged per mouse. Bars represent mean + SEM; n = 8 in the control groups and n = 6 in the bleomycin-treated groups. Statistical significance of the results was calculated by two-way ANOVA followed by Tukey’s multiple comparisons test.

To begin to assess whether MKP-1 might play a role in scleroderma-like skin fibrosis, dermal thickness was evaluated in wild-type and MKP-1-deficient mice following local bleomycin injections. As shown in Figure 1, bleomycin treatment increased dermal thickness in wild-type mice, and it was even further increased in MKP-1-deficient mice (Figure 1A,C). We weighed standard size full-thickness skin samples (6 mm in diameter) and observed that genetic deletion of MKP-1 also resulted in significantly more increased skin weight in bleomycin-treated mice compared to wild-type mice (Figure 1B). In addition, the subcutaneous fat layer was decreased in bleomycin-injected skin from both genotypes, and an interaction between the genotype and bleomycin was seen suggesting a more pronounced effect in the MKP-1-deficient mice (Figure 1A,D).

### 2.2. Collagen Deposition and Expression Is Increased in Bleomycin-Treated Skin from MKP-1-Deficient Mice

**Figure 2:**
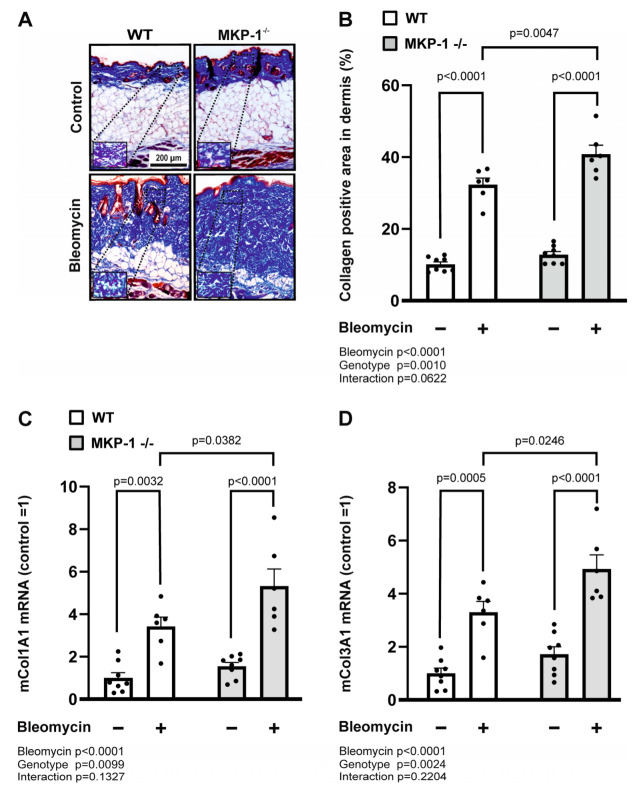
Collagen content and expression are increased in bleomycin-treated skin from MKP-1-deficient mice. (**A**) Representative images of Masson’s trichrome-stained sections of control skin and skin treated with bleomycin from wild-type (WT) and MKP-1-deficient (MKP-1^−/−^) mice are shown. Original magnification 10×, magnification in insets 20×, scale bar 200 µm. (**B**) Collagen content (% of collagen positive area in dermis was measured using Image J program. (**C**,**D**) Collagen 1A1 (Col1A1; (**C**)) and collagen 3A1 (Col3A1; (**D**)) mRNA expression in control skin and in skin treated with bleomycin from wild-type (WT) and MKP-1-deficient (MKP-1^−/−^) mice was measured by quantitative RT-PCR analysis and normalized against GAPDH mRNA. Col1A1 and Col3A1 levels in WT control skin was set as 1, and the other values are given in relation to that value. Bars represent mean + SEM; n = 8 in the control groups and n = 6 in the bleomycin-treated groups. Statistical significance of the results was calculated by two-way ANOVA followed by Tukey’s multiple comparisons test.

To probe whether bleomycin-induced increased dermal thickness in MKP-1-deficient mice corresponds with increased extracellular matrix deposition (ECM), we investigated the collagen content and collagen expression in the dermal skin samples from wild-type and MKP-1-deficient mice following bleomycin injections. For direct visualization of collagen and histological assessment of collagen deposition, Masson’s trichrome stain was utilized. Both wild-type and MKP-1-deficient mice responded to bleomycin treatment with an increase in collagen content in the skin. Bleomycin injections in MKP-1-deficient mice resulted in a significantly higher collagen accumulation in dermis compared to the wild-type mice (Figure 2A,B).

Next, we studied the expression of collagens 1A1 and 3A1 by qRT-PCR in skin samples from wild-type and MKP-1-deficient mice following bleomycin injections. As shown in Figure 2, bleomycin treatment increased dermal collagen 1A1 and collagen 3A1 expression in both wild-type and MKP-1-deficient mice, and the collagen expression levels were significantly higher in MKP-1-deficient mice (Figure 2C,D).

### 2.3. Expression of Profibrotic Factors and Chemokines Is Enhanced in Bleomycin-Treated Skin from MKP-1-deficient Mice

**Figure 3:**
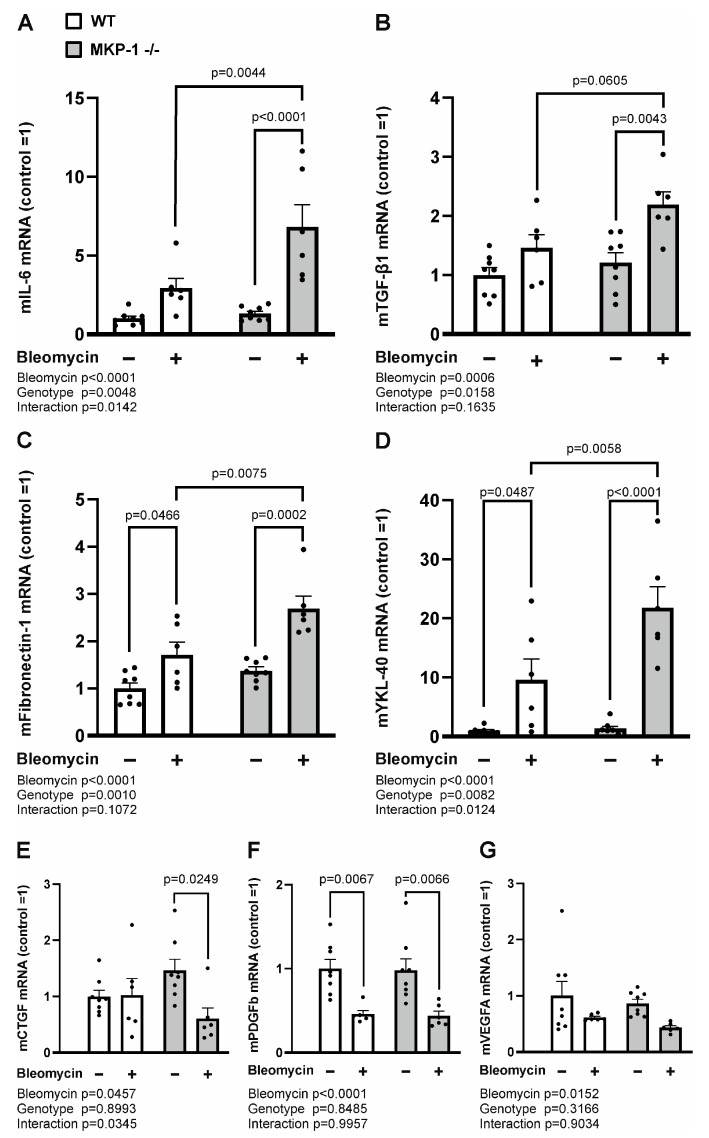
Expression of IL-6, TGF-β1, fibronectin-1 and YKL-40 is increased in bleomycin-treated skin from MKP-1-deficient mice. IL-6 (**A**), TGF-β1 (**B**), fibronectin-1 (**C**), YKL-40 (**D**), CTGF (**E**), PDGFB (**F**) and VEGFA (**G**) mRNA expression in untreated control skin and in the skin treated with bleomycin from wild-type (WT) and MKP-1-deficient (MKP-1^−/−^) mice was measured by quantitative RT-PCR analysis and normalized against GAPDH mRNA. IL-6, TGF-β1, fibronectin-1, YKL-40, CTGF, PDGFB and VEGFA levels in WT control skin was set as 1, and the other values are given in relation to that value. Bars represent mean + SEM; n = 8 in the control groups and n = 6 in the bleomycin groups. Statistical significance of the results was calculated by two-way ANOVA followed by Tukey’s multiple comparisons test.

After having detected that fibrosis was increased in bleomycin-treated skin from MKP-1-deficient mice, we investigated the expression of fibrogenesis mediators interleukin-6 (IL-6), transforming growth factor-β1 (TGF-β1) and fibronectin-1. We observed an increase in these mediators in bleomycin-treated skin more pronouncedly in MKP-1-deficient than in wild-type mice (Figure 3A–C).

YKL-40, also named chitinase-3-like protein-1 (Chi3L1), has been shown to be associated with inflammatory processes and increased fibrotic activity. Accordingly, YKL-40 expression was found to be upregulated in bleomycin-treated skin from both wild-type and MKP-1-deficient mice compared to control skin. Interestingly, the bleomycin-enhanced YKL-40 expression was higher in the skin samples from MKP-1-deficient mice than in those from wild-type mice (Figure 3D). However, no difference was found in connective tissue growth factor (CTGF), platelet-derived growth factor subunit B (PDGFB), or vascular endothelial growth factor A (VEGFA) expression between wild-type and MKP-1-deficient mice skin treated with bleomycin (Figure 3E–G).

**Figure 4:**
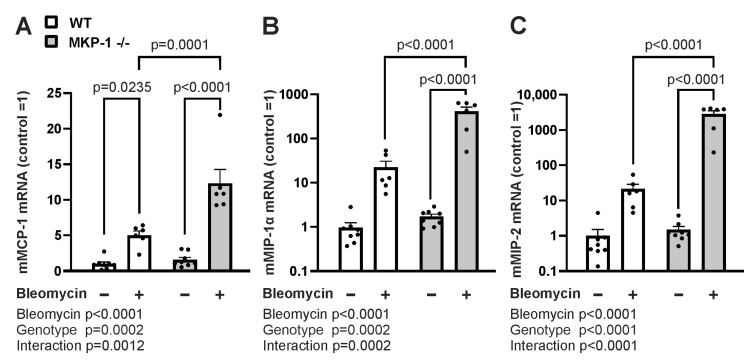
Chemokine expression is increased in bleomycin-treated skin from MKP-1-deficient mice. MCP-1 (**A**), MIP-1α (**B**), and MIP-2 (**C**) mRNA expression in untreated control skin and in the skin treated with bleomycin from wild-type (WT) and MKP-1-deficient (MKP-1^−/−^) mice was measured by quantitative RT-PCR analysis and normalized against GAPDH mRNA. MCP-1, MIP-1α and MIP-2 levels in WT control skin was set as 1, and the other values are given in relation to that value. Bars represent mean + SEM; n = 8 in the control groups and n = 6 in the bleomycin groups. Statistical significance of the results was calculated by two-way ANOVA followed by Tukey’s multiple comparisons test.

Chemokines together with cytokines contribute to the development of fibrosis through the recruitment of collagen producing myofibroblasts and other important effectors cells to the site of injury. We analyzed the expression of three important chemokines implicated in the fibrotic process: monocyte chemoattractant protein-1 (MCP-1), macrophage inflammatory protein-1 alpha (MIP-1α) and macrophage inflammatory protein-2 (MIP-2). Bleomycin treatment induced increased chemokine expression in both wild-type and MKP-1-deficient mice, but the response was higher in MKP-1-deficient mice (Figure 4).

## 3. Discussion

Our results show, for the first time, that MKP-1-deficient mice treated with bleomycin present a significant increase in the dermal thickness and lipodystrophy as well as in the gene expression of inflammatory and profibrotic factors as compared to wild-type mice. The present findings support our hypothesis that MKP-1 has a protective role in the fibrotic response induced by bleomycin and known to mimic scleroderma. Understanding the pathogenesis of scleroderma, and especially its early features, is essential to discover novel drug targets to treat scleroderma and fibrosis.

Bleomycin-induced dermal fibrosis is considered as an accurate experimental model for the study of scleroderma [20]. It reproduces the early stages of the disease characterized by vasculopathy, autoimmunity and inflammation with subsequently increased production and accumulation of collagen and other ECM components into the dermis and replacement of the adipose layer by fibrotic tissue (lipodystrophy). The accumulation of the collagen and other ECM proteins lead to increased skin thickness and fibrosis [21]. Accordingly, we observed that bleomycin treatment resulted in a significant increase in dermal collagen content in wild-type mice. The findings on protective role of MKP-1 in this process were supported by the results, where MKP-1-deficient mice had higher collagen deposition and increased expression of collagens 1A1 and 3A1 and fibronectin-1 in dermis as compared to wild-type animals.

Accumulating evidence indicates that inflammatory response is necessarily preceding fibrogenesis [1,2]. Several studies have reported a high number of infiltrating activated macrophages and lymphocytes in the skin of patients with scleroderma [22,23]. These inflammatory cells are key producers of a variety of profibrotic cytokines, such as TGF-β and IL-6. TGF-β is a potent inducer of ECM production and a key factor in fibrogenesis in different fibrosing diseases [21,22], and IL-6 is an important regulator of fibroblasts activation [22,24]. Therefore, we measured TGF-β and IL-6 expression in the skin from wild-type and MKP-1-deficient mice treated with bleomycin. Interestingly, MKP-1 deletion resulted in an increase in the expression of both cytokines, supporting a role for this nuclear phosphatase in the pathogenesis of the bleomycin-induced dermal fibrosis. We also analyzed the expression of YKL-40, a chitinase-3-like protein-1 (Chi3L1), that is upregulated in many pathological and inflammatory conditions [25,26,27,28] and associated with increased fibrotic activity [7,29,30]. YKL-40 may also actively promote fibrosis as it induces apoptosis of classically activated M1 macrophages, but not alternatively activated M2 macrophages, which are known to possess profibrotic properties [31,32]. Here, we demonstrated that YKL-40 expression was regulated by MKP-1 as the levels were enhanced in the skin from bleomycin-treated mice with MKP-1 genetic deletion when compared with wild-type counterparts.

Chemokines are leukocyte chemoattractant that cooperate with profibrotic cytokines in the development of fibrosis by recruiting collagen-producing myofibroblasts, macrophages and other key effector cells to sites of tissue injury. A large number of chemokines are involved in the mechanism of fibrogenesis, but the CC- and CXC-chemokine families have exhibited important regulatory roles [33]. CCL-2/MCP-1, CCL-3/MIP-1α and CXCL2/MIP-2 are examples of chemokines identified as profibrotic mediators [34,35]. Accordingly, we showed here that chemokines MIP-1α, MIP-2 and MCP-1 were increased in response to bleomycin treatment and importantly, the expression of these chemokines was strongly upregulated in MKP-1-deficient mice compared to wild-type counterparts. This finding further supports the protective role for MKP-1 in controlling migration of profibrotic inflammatory cells to the site. Moreover, we did not detect significant differences in control groups between wild-type and MKP-1-deficient mice, suggesting that MKP-1 deficiency increases fibrogenesis only after an appropriate trigger.

Considering the present findings on the protective role of MKP-1 in an experimentally induced scleroderma, it is of interest that MKP-1 has been shown to increase IL-12 production [18,19]. IL-12 promotes Th1 response, which is thought to prevent from Th2 type response typical for the development of scleroderma [2,18,19]. Interestingly, increased circulating IL-12 concentrations have been reported in patients with scleroderma, particularly in the healing phase of the disease [36]. In addition, cases of scleroderma such as morphea skin lesions have been reported in psoriasis patients using ustekinumab, an antagonist of IL-12 and IL-23 [37,38]. However, further studies are needed to understand the detailed mechanisms how MPK-1 downregulates the pathogenesis of scleroderma.

In the light of the present results, drugs inducing MKP-1 could have antifibrotic effects in scleroderma and fibrosis. Drugs known to induce MKP-1 include antirheumatic gold compounds, aurothiomalate and auranofin [39], phosphodiesterase (PDE)4 inhibitor rolipram [15,40], β2-agonists [41,42] and glucocorticoids (GCs) [11,15,43,44,45]. GCs are used as a part of the drug treatment in SSc-related diffuse cutaneous disease, interstitial lung disease, and inflammatory arthritis, although their efficacy is limited and the risk of renal adverse effects restrains their use in SSc patients [46]. Potential beneficial effect of GCs on SSc via increased MKP-1 expression may be complicated by their widespread effect on the expression of hundreds of genes involved, e.g., in extracellular matrix organization and cell metabolism [47]. cGMP is an intracellular signaling molecule that has been reported to increase MKP-1 expression [48], and cellular cGMP levels are regulated by the activity of the enzymes guanylate cyclase (GC) and by phosphodiesterase 5 (PDE5). The current findings on the role of MKP-1 in bleomycin-induced fibrosis also shed light on the hitherto unknown mechanisms of the antifibrotic effects of cGMP-enhancers, namely the sGC stimulator riociguat and PDE5 inhibitors, recommended currently to the treatment of SSc-related disease subtypes [46].

To our knowledge, MKP-1 has not been investigated in patients with scleroderma, and this is the first study in an experimental model of the disease. A limitation of the applicability of the current findings to the clinical disease lies in the potential differences in the pathogenesis of the bleomycin model and the actual human disease. Furthermore, the detailed mechanisms how MKP-1 downregulates or retards the pathogenesis of scleroderma remains to be investigated.

Although MKP-1 has not directly been studies in scleroderma, it is of interest that inhibitors of the MAP kinases p38 and JNK were reported to have antifibrotic effects in human SSc fibroblasts [49,50]. Furthermore, Ihn et al. also reported a constitutive phosphorylation and activation of p38 in SSc patient-derived fibroblasts. Considering that MKP-1 dephosphorylates and thereby inactivates MAP kinases p38 and JNK, those findings are in line with our results supporting their significance and applicability in SSc patients. Intriguingly, MKP-1 has also been identified as a primary candidate in a meta-analysis combining microarray data from patients with systemic sclerosis and chronic graft-versus-host disease—two diseases with common fibrotic skin and internal organ involvement [51]. Our present findings together with the cited data in the literature strongly encourage researchers to continue to investigate the potential role of MKP-1 as a factor and drug target in SSc.

In conclusion, we have demonstrated for the first time that MKP-1 deficiency in mice promotes skin fibrosis by augmenting profibrotic and proinflammatory factors, suggesting a potential protective role of MKP-1 in fibrosing diseases. At the moment, the treatment modalities for scleroderma and other fibrosing diseases are limited. The present study introduces MKP-1 as a potential new treatment target for scleroderma and compounds able to increase the expression/activity of MKP-1 as potential new drugs for the treatment of fibrosing pathologies.

## 4. Materials and Methods

### 4.1. Animals

MKP-1-deficient male C57BL/6 mice and corresponding wild-type controls were used in the bleomycin-induced model of scleroderma. The MKP-1-deficient mice were originally generated at Bristol-Myers Squibb Pharmaceutical Research Institute (Princeton, NJ, USA) [52]. Mice were bred under standard conditions (12h:12h light: dark cycle, +22 ± 1 °C temperature, 50–60% humidity), and water and food were constantly available *ad libitum*. Experimental procedures were performed according to the legislation on the protection of animals used for scientific purposes (Directive 2010/63/EU), and the license for the experiment was approved by National Animal Experiment Board (ESAVI/10109/04.10.07/2015).

### 4.2. Bleomycin Treatment

Bleomycin (Cayman Chemical, Ann Arbor, MI, USA) was dissolved in sterile phosphate-buffered saline (PBS) and diluted to 0.5 mg/mL. The upper dorsa of mice were shaved, and a square (about 1.5 cm^2^) was drawn with a marker. Sevoflurane inhalation was used to anesthetize mice, and 100 μL of bleomycin was administered by using a 27-gauge needle into the shaved area rotating injection sites, every other day for 4 weeks. On the day following the last injection, CO_2_ was used to euthanize the mice, and punch biopsies of 6 mm diameter were taken from the injected skin. Two skin biopsy specimens were weighed and fixed in 10% formalin and used for histological analyses. One specimen was stored in RNAlater solution (Invitrogen, Life technologies, Carlsbad, CA, USA) and processed for RNA extraction. Lesioned skin samples were obtained from age-matched male MKP-1-deficient and wild-type mice (n = 6) injected with bleomycin. Control skin was collected from MKP-1-deficient and wild-type mice (n = 8) that did not receive bleomycin treatment.

### 4.3. Histological Analysis

The skin specimens were fixed in 10% formalin, embedded in paraffin, cut in 6 μm sections, and mounted on slide. Hematoxylin and Eosin (HE; Histolab Products AB, Göteborg, Sweden) or Masson’s trichrome (Sigma-Aldrich Chemical Company, St. Louis, MO, USA) were used to stain slides. Dermal and adipose tissue thickness (μm) were measured in HE-stained sections at six randomly selected locations in each section using the Image J program. Collagen accumulation (% of total area) was measured in Masson’s-stained sections by Image J program as previously described [53].

### 4.4. RNA Extraction and Quantitative RT-PCR

RNA from skin samples was extracted using the GenElute Mammalian Total RNA Miniprep Kit (Sigma-Aldrich Chemical Company, St. Louis, MO, USA), according to the manufacturer’s instructions. Total RNA was reverse-transcribed to cDNA using Maxima First Strand cDNA Synthesis Kit for RT-qPCR (Thermo Fisher Scientific, Waltham, MA, USA). After the transcription reaction, the cDNA obtained was subjected to PCR using TaqMan Universal PCR Master Mix and ABI PRISM 7500 Sequence detection system (Applied Biosystems). The primer and probe sequences and concentrations were optimized according to the manufacturer’s guidelines in TaqMan Universal PCR Master Mix Protocol part number 4304449 revision C and were 5′-GCATGGCCTTCCGTGTTC-3′ (mouse glyceraldehyde-3-phosphate dehydrogenase (GAPDH) forward primer, 300 nM), 5′-GATGTCATCATACTTGGCAGGTTT-3′ (mouse GAPDH reverse primer, 300 nM), 5′-TCGTGGATCTGACGTGCCGCC-3′ (mouse GAPDH probe, 150 nM, containing 6-FAM as 5′-reporter dye and TAMRA as 3′-quencher) and 5′-TCGGAGGCTTAATTACACATGTTC-3′ (mouse interleukin-6 (IL-6) forward primer, 900 nM), 5′-CAAGTGCATCATCGTTGTTCATAC-3′ (mouse IL-6 reverse primer, 300 nM), 5′-CAGAATTGCCATTGCACAACTCTTTTCTCA-3′ (mouse IL-6 probe, 200 nM, containing 6-FAM as 5′-reporter dye and TAMRA as 3′-quencher). Primers and probes were purchased from Metabion (Martinsried, Germany). TaqMan Gene Expression assays for mouse COL1A1 (Mm00801666_g1), mouse COL3A1 (Mm01254476_m1), mouse TGF-β1 (Mm01178820_m1), mouse fibronectin-1 (Mm01256744_m1), mouse CTGF (Mm01192933_g1), mouse PDGFB (Mm00440677_m1), mouse VEGFA (Mm01281449_m1), mouse MCP-1 (Mm00441242_m1), mouse MIP-1α (Mm99999057_m1), mouse MIP-2 (Mm00436450_m1) and mouse YKL-40 (Mm00801477_m1) were obtained from Thermo Fisher Scientific, Waltham, MA, USA.

The PCR cycling parameters were as follows: incubation at 50 °C for 2 min, incubation at 95 °C for 10 min, and thereafter 40 cycles of denaturation at 95 °C for 15 s and annealing and extension at 60 °C for 1 min. The mRNA levels were normalized against the housekeeping gene GAPDH mRNA levels and quantified using the ΔΔCt method.

### 4.5. Statistical Analysis

The results are presented as the mean + standard error of the mean (SEM). Two-way analysis of variance ANOVA followed by Tukey’s multiple comparison test was used. p values less than 0.05 were considered significant. Data were analyzed using the Prism computerized package (Graph Pad Software, San Diego, CA, USA).

## Data Availability

All relevant data is included in the manuscript.
